# Vitamin D Receptor Polymorphisms and Non-Melanoma Skin Cancer Risk: A Case-Control Study

**DOI:** 10.3390/jcm9123819

**Published:** 2020-11-25

**Authors:** Carolina Morgado-Águila, Purificación Rey-Sánchez, Guadalupe Gil-Fernández, María Carmen Costa-Fernández, Francisco José Rodríguez-Velasco

**Affiliations:** 1 Department of Plastic and Reconstructive Surgery, Cáceres University Hospital Complex, Cáceres, 10001 Extremadura, Spain; carolina.morgado@salud-juntaex.es; 2 Department of Nursing, Faculty of Nursing and Occupational Therapy, University of Extremadura, Cáceres, 10003 Extremadura, Spain; mccosta@unex.es; 3 Department of Nursing, Faculty of Medicine, University of Extremadura, Badajoz, 06006 Extremadura, Spain

**Keywords:** vitamin D3 receptors, squamous cell neoplasms, basal cell carcinoma, skin cancers

## Abstract

Exposure to sunlight is the major source of vitamin D and the main environmental cause of non-melanocytic skin cancers. Vitamin D, partly mediated through the vitamin D receptor (VDR), has potential therapeutic applications in skin cancer. The aim of this study was to investigate the association of *BsmI* and *ApaI* VDR polymorphisms among patients with non-melanoma cancers and controls. An observational case-control study was conducted in a sample of 154 subjects. We observed no significant effects between these polymorphisms and skin cancer risk. When stratified for gender, GG and AG *BsmI* polymorphisms significantly increased the risk of basal cell carcinomas in males. In relation to *ApaI*, all three polymorphisms significantly increased the risk of basal cell carcinoma in males. When stratified for age, we found that being 70 years of age or younger was a protective factor against both skin cancers. Being a female and 70 years old or younger was a protective factor for basal cell carcinoma. A comparison of the frequencies of the VDR genotypes in patients older than 70 years vs. 70 years or younger also revealed age-dependent variations in patients with non-melanoma skin cancer. Our study suggests a role for VDR polymorphisms in non-melanoma skin cancer development.

## 1. Introduction

In addition, to bone mineralization and maintenance of calcium balance, 1α25(OH)_2_D_3_ exerts physiological functions, including the regulation of growth and differentiation in a broad variety of normal and malignant cells [1,2].

The major source of vitamin D for most humans is exposure to sunlight [3]. It is known that vitamin D requires two obligate hydroxylations, first in the liver and then in the kidney, to create the active form of vitamin D, 1α25(OH)_2_D_3_. Most tissues and cells in the body, including skin, have nuclear receptors for 1α25(OH)_2_D_3_, called vitamin D receptors (VDRs). The VDR is an intracellular hormone receptor that specifically binds the active form of vitamin D and interacts with target-cell nuclei to produce a variety of biological effects. Upon ligand activation, VDR binds specific nucleotide sequences in target genes to activate or repress their expression.

An increasing incidence of skin cancer has been described in all light-skinned populations and to some extent in Asian and South American populations [4]. These increases concern all types of skin cancer, including basal cell carcinoma (BCC), squamous cell carcinoma (SCC) and cutaneous malignant melanoma. It is widely accepted that sun exposure is the main environmental cause of cutaneous melanoma and of non-melanocytic skin cancer, BCC and SCC. Likewise, in many populations, the level of vitamin D in individuals is also related to their sun exposure [5].

Ultraviolet B radiation appears to be largely responsible for the induction of non-melanocytic skin cancer, and its action spectrum is similar to that needed for the synthesis of vitamin D. However, exposure to UVB increases endogenous vitamin D synthesis; notably, the most intriguing, important and unappreciated biologic functions of 1α25(OH)_2_D_3_ is its ability to downregulate hyperproliferative cell growth.

This paradoxical effect can be explained by evidence suggesting that skin synthesis of vitamin D is self-limited and, in light-skinned people, vitamin D synthesis activity fades away after five to ten minutes. Longer durations of sun exposure will not further increase vitamin D but will increase skin cancer risk [5,6].

Anti-neoplastic actions of 1α25(OH)_2_D_3_ are partly mediated through the VDR. The physiological and pharmacological actions of 1α25(OH)_2_D_3_ in various cells have indicated potential therapeutic applications of VDR ligands in cancers.

The VDR gene is located on chromosome 12q12-q14. Several single nucleotide polymorphisms (SNPs) have been identified. We studied two of these polymorphisms located at the 3′-end of the gene, defined by the restriction enzymes *BsmI* (rs1544410) and *ApaI* (rs7975232). The *BsmI* polymorphism is located in intron 8 at the 3′ end of the VDR gene. In this polymorphism, the nucleotide adenine is substituted with guanine (G > A). As a silent SNP, it does not change the amino acid sequence of the encoded protein, yet it may affect gene expression by regulating mRNA stability [7]. Like *BsmI*, the *ApaI* polymorphism is located at the 3′ end of the VDR gene in intron 8 and causes guanine nucleotides to be substituted with thymine (T > G) [8].

Frequencies of the VDR genetic variants differ according to ethnic groups [9]. These polymorphisms modulate the activity of the VDR. It has been hypothesized that a less-active VDR could be associated with either an increased susceptibility to cancer risk or a more aggressive disease [5].

Many studies have investigated the association of one or several VDR polymorphisms with cancer risk, mostly for prostate, breast and colon cancer, with conflicting results in different populations [10]. Fewer studies have investigated the association of these VDR polymorphisms with non-melanoma skin cancer risk [11].

This study has assessed the possible implications of the *BsmI* and *ApaI* VDR polymorphisms for SCC and BCC prevalence in the Spanish Caucasian population.

## 2. Experimental Section

### 2.1. Study Design, Setting and Participants

An observational case-control study was conducted at the Plastic Surgery and Dermatology Service of the Complejo Hospitalario Universitario de Cáceres (the only reference Center in Extremadura for Plastic Surgery—Spain). We aimed to have sufficient statistical power to detect medium effect sizes (anticipated Cohen’s *d* = 0.61/OR = 3) with anticipated proportion of exposed in the case group (p2) = 0.60, β = 0.90 and α = 0.05, which required a minimum sample size of 144 participants [12]. A total of 172 subjects were initially evaluated. The patient group consisted of 99, randomly selected, Caucasian subjects with suspected non-melanoma skin cancer diagnosed in the Dermatology or Plastic Surgery Department in Cáceres (Spain), between 2016 and 2017. Randomization was performed using SPSS software to generate random numbers (IBM Corporation, Armonk, NY, USA). Additionally, 73 healthy Caucasian individuals were enrolled to serve as controls after examination by an experienced plastic surgeon confirmed that they did not have any type of skin cancer. Furthermore, given that gender and age are variables related to the occurrence of the disease and that these variables were intended to be studied, the criterion of random selection of subjects was adopted (with the same inclusion and exclusion criteria in both groups), avoiding selective pressure on it (selection bias).

The cases were recruited at the first clinical attendance, where surgery was proposed. Approximately 2 weeks after this first visit, at the operating room, peripheral venous blood samples were drawn and the suspected skin cancers were surgically excised. The controls were recruited at the first clinical attendance when visiting the facilities for other medical consultations, such as traumatic wounds, and blood samples were collected at that time. Both cases and controls come from rural areas and they have similar sun exposure habits. Specifically, they all come from Extremadura, a mostly rural region in Spain in which we have a high solar radiation (Latitude 39°). Even though these skin malignancies occur mostly in photo-exposed areas, controls were thoroughly examined—exposed and non-exposed areas—and it was confirmed that they did not have any type of skin cancer.

**Figure 1:**
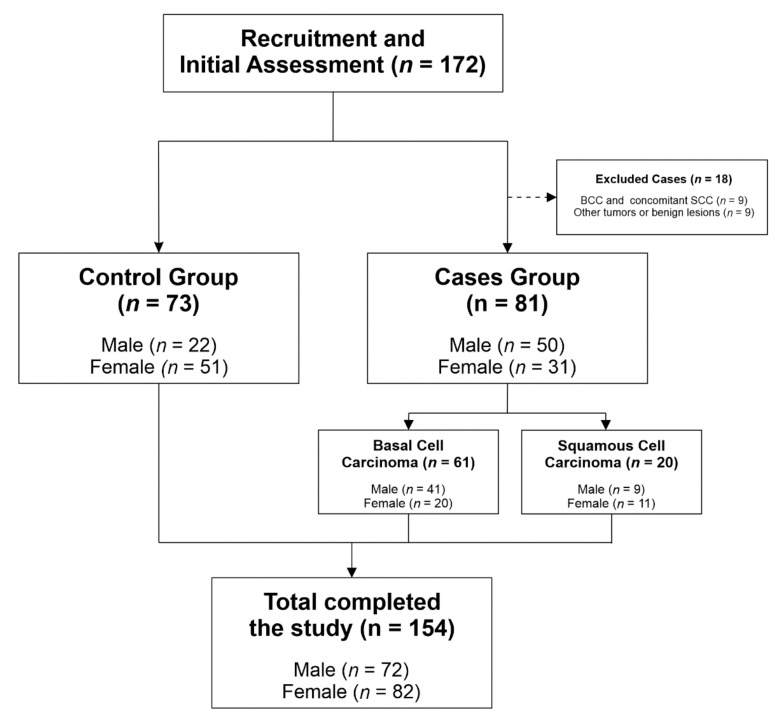
Participant selection process.

Only patients with BCC or SCC were included. We found 61 patients with BCC and 20 with SCC. If non-malignant lesions or other types of skin cancer were encountered, we excluded these patients from our study (9 patients). We also excluded 9 patients who had or previously had both skin cancers (BCC and SCC). None of the included patients was an organ transplant recipient, none was being treated with immunosuppressive drugs, was under 18 years of age or took vitamin complexes. For our sample size (*n* control = 73 and *n* cases = 81), we calculated the statistical power to detect medium effect sizes (anticipated Cohen’s *d* = 0.61/OR = 3) with p2 = 0.60 and α = 0.05, obtaining an output of 0.91. Figure 1 illustrates the participant selection process.

We chose to divide the age variable according to the closest cut-off value in relation to the Mean (68.22) and Median (68.50) of that variable in our study sample, which is 70 years old (≤70 age/>70 age).

Each subject, case or control, gave written informed consent before entering the study, which met the requirements of the World Medical Association Declaration of Helsinki. The study had previously been approved by the local Ethics Committee and the Internal Review Board (IRB) of the University of Extremadura (protocol ID number: 74/2015). This study was reported following the Strengthening the Reporting of Observational Studies in Epidemiology (STROBE) guidelines for reporting observational studies [13].

### 2.2. Selection of Polymorphisms and Genotyping

The occurrences in blood samples of the two VDR polymorphisms were assessed in patients with non-melanoma skin cancer and controls by an independent laboratory (STAB—Servicio de Técnicas Aplicadas a la Biociencia) in the nearby city of Badajoz (Spain). The laboratory personnel did not know the objective of our study and were blinded to the sample status (case or control). Quality control samples were inserted to validate genotyping procedures, and concordance for the blinded samples was 100%.

Genotyping was performed with the TaqMan^TM^ Genotyping Master Mix (Thermo Fisher Scientific Inc, Applied Biosystems^TM^, Waltham, MA, USA) and the TaqMan^TM^ SNP Genotyping Assays rs1544410 and rs7975232 (Applied Biosystems) using the QuantStudio 6 Flex platform (Applied Biosystems) and the software QuantStudio Real-Time PCR.

The *BsmI* alleles (rs1544410) were identified using the sequence AGCAGAGCCTGAGTATTGGGAATG[C/T]GCAGGCCTGTCTGTGGCCCCAGGAA, and the *ApaI* alleles (rs7975232) were identified using AAGGCACAGGAGCTCTCAGCTGGGC[A/C]CCTCACTGCTCAATCCCACCACCCC.

All other information about primers, probes and conditions for genotyping assays is available upon request.

### 2.3. Skin Samples

Surgical excision was always performed by the same surgeon according to protocols [14,15], and the samples were sent to the Pathology Laboratory for Histologic Study in a tertiary-level hospital. Laboratory personnel studied the samples as part of their routine work, and the personnel did not know that the patients were being studied for any reason other than clinical management.

### 2.4. Statistical Methods

We used a χ^2^ test to assess whether the genotypes for each of the polymorphisms were in Hardy–Weinberg Equilibrium (HWE).

The associations in allele frequencies between the affected and control populations were assessed by logistic regression analysis. The likelihood ratio test (LRT) was calculated to evaluate the heterogeneity in the effects of the genotypes on the different types of skin cancer.

Univariate and multivariate logistic regression analyses were performed to evaluate the association between independent variables (factors) and the occurrence of non-melanoma skin cancer (dependent variable). Multivariate and multinomial logistic regression was employed to calculate the odds ratios (OR) and 95% confidence intervals (IC) to assess the risk of skin cancer for genotypes, age and gender among the subjects.

The frequencies of the polymorphisms between the different groups were compared with the χ^2^ test or Fisher’s exact test. The significance cut-off value was *p* ≤ 0.05. The continuous variables are expressed as the mean ± standard deviation, and the categorical variables are expressed as percentages.

All statistical analyses were performed using SPSS for Windows, version 24.0 (IBM Corporation, Armonk, NY, USA).

## 3. Results

### 3.1. Descriptive Characteristics of Cases and Controls

A total of 154 subjects were included in our study. The patient group consisted of 81 subjects with non-melanoma skin cancer, and 73 healthy individuals were enrolled to serve as controls. A total of 72 (46.75%) were males, and 82 (53.24%) were females.

Our subjects were between 38 and 94 years old with a mean age of 68.22 ± 14.53 years (males: 70.69 ± 13.51; females: 66.05 ± 15.13). The mean age of the control subjects was 60.58 ± 12.90 years and that of the BCC and SCC cases were 74.03 ± 12.19 and 78.40 ± 12.66 years, respectively.

The distribution of the percentages of the genotypes (*BsmI* and *ApaI* polymorphism) of the analysed sample met HWE, both in the total sample (HWE *BsmI p* = 0.85 and HWE *ApaI p* = 0.51), in cases (HWE *BsmI p* = 0.48 and HWE *ApaI p* = 0.58) and in controls (HWE *BsmI p* = 0.29 and HWE *ApaI p* = 0.11). It is observed that the most frequent genotype for *BsmI* (both in the total sample and in cases and controls) was the *AG* (48.70%, 43.21% and 54.79%, respectively), however, the least frequent was the *AA* genotype (15.58%, 16.05% and 15.07%, respectively). The most frequent genotype for *ApaI* (both in the total sample and in cases and controls) was the GT (52.60%, 46.91% and 58.90%, respectively), while the least frequent was the GG genotype (22.08%, 25.93% and 17.81%, respectively).

### 3.2. VDR SNPs, Age and Gender and Non-Melanoma Skin Cancer Risk

Regarding the polymorphisms in the VDR gene, no significant main effects were observed between the *BsmI* or *ApaI* polymorphism genotypes and the skin cancer risk in the multinomial logistic regression model (Table 1).

However, if the *BsmI* and *ApaI* polymorphisms were stratified for gender and skin cancer risk, we found that the *GG* and *AG BsmI* polymorphisms significantly increased the risk of BCC in males (OR: 7.01; IC 95%: 2.35–20.92 and OR: 4.59; IC 95%: 1.60–13.18, respectively). The multivariate logistic regression model carried out was significant, taking [*BsmI* polymorphism] * [Gender] as factors (*p* = 0.00), and [*ApaI* polymorphism] * [Gender] (*p* = 0.00). In relation to *ApaI*, we observed that the three polymorphisms (*TT*, *GG* and *GT*) significantly increased the risk of BCC in males (OR: 3.40; IC 95%: 1.06–10.87, OR: 11.90; IC 95%: 3.19–44.37 and OR: 6.80; IC 95%: 2.34–19.75, respectively). Although all three polymorphisms increased the risk of BCC in males, they did so in different amounts depending on which polymorphism was involved. The *GG ApaI* polymorphism could increase the risk for BCC nearly 12 times. No significant main effects were observed for SCC in the analyses of the *ApaI* or *BsmI* polymorphisms (Table 2).

We also used the multinomial logistic regression test to assess whether age and gender were related to skin cancer risk. The male gender was significantly associated with an increased risk of BCC (OR: 4.38; IC 95%: 1.93–9.90) but not with SCC risk, compared to controls. It was also observed that being 70 years of age or younger was a protective factor against both BCC and SCC risk (OR: 0.12; IC 95%: 0.05–0.28 for BCC and OR: 0.07; IC 95%: 0.02–0.24 for SCC). There was no significant association between BCC or SCC risk related to VDR SNPs, age or gender (Table 1).

If we further stratified the cases and controls regarding the type of skin cancer, age and gender, we find that being a female and 70 years old or younger was a protective factor for BCC (OR: 0.12; IC 95%: 0.04–0.37). The multivariate logistic regression model carried out was significant, taking [Age] * [Gender] as factors (*p* = 0.00). (Table 3).

The comparison of the frequencies of the VDR genotypes in patients older than 70 years vs. those that were 70 years or younger revealed evidence of age-dependent variations in the patients with BCC or SCC. When stratifying age and type of skin cancer, we found that, except for the *GT ApaI* polymorphism, all the other polymorphisms were protective against BCC in the patients who were 70 years old or younger. Similarly, the *AG BsmI* and *GT ApaI* polymorphisms were protective against SCC risk in the patients who were 70 years old or younger. The multivariate logistic regression model carried out was significant, taking [*BsmI* polymorphism] * [Age] as factors (*p* = 0.00), and [*ApaI* polymorphism] * [Age] (*p* = 0.00) (Table 4).

### 3.3. The Combined BsmI/ApaI Genotypes in the Patients with BCC or SCC and the Controls

There was a trend towards a higher prevalence of the *AGGT*, *GGGG* and *AATT* combined polymorphisms in our population (Table 5).

We studied the combined *BsmI* and *ApaI* genotypes to assess whether there was an association between among these genotypes and the patient cancer risk (BCC, SCC, control), but we did not find any interactions between these nine combined polymorphisms and cancer risk.

## 4. Discussion

As far as we are aware, no previous studies have involved a Spanish population to assess the possible associations between VDR polymorphisms and non-melanoma skin cancer.

It is already known that non-melanoma skin cancer is related to age [16,17,18] and gender [19,20], and this statement is corroborated by our study. However, we also found new and interesting results in relation to VDR polymorphisms and skin cancer risk: No significant main effects were observed when the *BsmI* or *ApaI* polymorphisms were studied alone, but there were significant and interesting results when adjusted for stratum age and gender. These results are conflicting when compared to those of a few similar published studies.

Skin cancers are the most common cancer type in most white-skinned populations and are an important health concern because they impose a substantial burden on health care systems [21,22].

In relation to skin cancer, it is known that ultraviolet radiation (UVR) has strong carcinogenic effects on skin tissue, which makes it a strong risk factor for melanoma and non-melanoma skin cancer [23,24]. Although it is obvious that the relationship between UVR and skin cancer is more complex than for other types of cancer, there is evidence of a protective effect of vitamin D. 1α25(OH)_2_D_3_ has anti-proliferation and pro-differentiation effects in both melanocytes and cutaneous melanoma cells, mediated through the VDR [25].

As early as the 1980s, it was shown that the active form of vitamin D, 1α25(OH)_2_D_3_, could inhibit the proliferation of melanoma cells [26]. Following these studies, the antiproliferative effects of 1α25(OH)_2_D_3_ have been demonstrated in a wide variety of cancer cell lines, including breast or prostate cancer lines [10].

It has been shown that vitamin D inhibits hedgehog signalling and proliferation in murine BCCs [27]. It has also been shown that topical vitamin D, acting via its hedgehog-inhibiting mechanism, may hold promise as an effective anti-BCC agent [27].

VDR is a crucial mediator of the cellular effects of vitamin D. A vast amount of information has been collected over the years regarding the association of VDR polymorphisms with the susceptibility of individuals to suffer from different diseases, such as cancer [10,28].

Associations of some of the VDR genotypes with skin cancer risk have been studied, both for melanoma [29,30,31,32] and non-melanoma skin cancer [33,34,35,36,37,38].

In 2007, Han et al. [34] observed that the *BsmI BB* genotype was significantly associated with an increased risk of SCC (OR: 1.51; IC 95%: 1.00–2.28) but not with BCC risk. An interaction was also observed between the *BsmI* polymorphism and the SCC risk among women with a high constitutional susceptibility, namely, lighter hair, fair skin colour, greater tendency to burn and more moles (*BB* versus *bb* OR: 1.85; IC 95%: 1.12–3.06; the *p*-value for this trend across the three genotype categories, *BB*, *Bb*, and *bb*, was 0.03). Moreover, they observed an interaction between the *BsmI* polymorphism and the total vitamin D intake on the SCC risk, suggesting that women with the BB genotype and high vitamin D intake have the highest risk of SCC.

If we consider solar keratoses as in situ SCC [39], we need to mention the study that Carless et al. [33] conducted to assess the association between solar keratoses and polymorphisms of the VDR gene. They found a significant difference in the genotype frequencies of the *TaqI* polymorphisms between affected and unaffected populations. The *TT/tt* genotype group was associated with a twofold increase in the odds of being affected by one or more solar keratoses. They did not observe an association between the *ApaI* polymorphism and solar keratosis prevalence when the genotypes were analysed alone. However, they did find a significant association when both polymorphisms were analysed in relation to other variables, such as skin colour or propensity to burn and/or tan. The fair-skinned people with the *ApaI AA/aa* genotypes had an approximately eightfold increase in the odds of being affected by solar keratoses compared with a fivefold increase in individuals with the *Aa* genotype and fair skin.

Later, in 2011, Lesiak et al. [36] studied a Polish population in search of a possible association between BCC and polymorphisms in the VDR gene. They did not find an association between *ApaI*, *TaqI* or *BsmI* and BCC. They found an association between the presence of the *TT* genotype in the *FokI* VDR polymorphism, resulting in a >10-fold higher risk of BCC development.

Köstner et al. [35] studied a German population to assess the possible association between *ApaI*, *BglI* and *TaqI* and BCC or SCC. There was a trend towards a higher distribution of homozygous genotypes for all three polymorphisms in the controls than in the BCC patients, but these differences were not statistically significant. No significant differences in the frequency of the individual genotypes between the SCC patients and the controls were found either. When the combined *ApaI/TaqI/BglI* genotype was analysed, an association of the genotype *AaTtBb* with BCC risk was found. They also found that the *aaTTbb* genotype was more frequent in the BCC and SCC groups compared to that of the controls.

Von Schuckmann et al. [37] found that the *ApaI* (rs7975232) *GG* + *GT* dominant genotypes were associated with a decreased occurrence of BCC when compared with the *TT* genotype (OR: 0.67; IC 95%: 0.46–0.96; *p* = 0.03). Nevertheless, the associations were no longer statistically significant in the adjusted model and after correction for multiple testing within each gene. Additionally, the SCC risk was decreased in participants with the *FokI* (rs2228570) *TT* vs. *CC* + *CT* genotypes, and this remained statistically significant after adjusting for confounders and correcting for multiple testing (ORadj: 0.34; CI 95%: 0.17–0.68; *p* = 0.00).

Burns et al. [40] found that participants with the *BsmI* SNP were twice as likely to develop non-melanoma skin cancer than participants with no mutation. Moreover, individuals with the A genotypes were only about half as likely to develop non-melanoma skin cancer. However, after adjusting for age and gender in the model, they did not find statistically significant results.

Very recent studies have followed this long-standing yet innovative line of research, as promising results are being found [38,41].

In our observational case-control study, no significant main effects were observed between the *BsmI* or *ApaI* polymorphism genotypes and skin cancer risk.

When stratified for gender and skin cancer risk, we found that GG and AG *BsmI* polymorphisms significantly increased the risk of BCC in males (OR: 7.01; IC 95%: 2.35–20.92 and OR: 4.59; IC 95%: 1.60–13.18, respectively). All the *BsmI* polymorphisms were protective against BCC in patients who were 70 years of age or younger. The *AG BsmI* polymorphism was also protective against SCC for the same age range.

Like Lesiak and colleagues and Von Schuckmann and colleagues, our findings showed no statistically significant associations between the *BsmI* polymorphism genotypes and cancer risk. Han and colleagues observed that the *BsmI* dominant-*BB* (-GG) genotype was significantly associated with an increased risk of SCC, but our study did not support their findings.

On the other hand, Von Schuckmann and colleagues found that *ApaI* (rs7975232) *GG* and *GT* genotypes were associated with a decreased occurrence of BCC, but these associations were no longer statistically significant in the adjusted model. The same happened to Burns et al. [40] who found that individuals with the A genotypes were half as likely to develop non-melanoma skin cancer, but the associations were no longer statistically significant in the adjusted model.

Like our findings, none of the other authors that had previously studied the *ApaI* polymorphisms observed any association between this polymorphism and non-melanoma skin cancer. As mentioned before, we found an association between the *ApaI* polymorphisms, BCC and SCC when analyses were adjusted for stratum gender and age. Similarly, Carless and colleagues found an association when they analysed their results in relation to other variables, such as skin colour or propensity to burn and/or tan.

We found no significant association between the combined genotypes and skin cancer, most likely due to the relatively low case numbers.

It should be noted that individual VDR polymorphisms cannot be regarded as independent prognostic factors [11]. We did not take plasma measurements of vitamin D, which is a limitation of our study. If not vitamin D plasma levels, at least interactions that determine vitamin D status, such as diet or supplements, solar UV exposure or body mass index, should be determined in future studies to confirm the links between the gene polymorphisms and BCC and SCC in our population.

Within the limited published data, there is no evidence that any polymorphism is clearly involved in the risk of having BCC or SCC. The inconsistent results may be due to high heterogeneity between studies and populations. This present study, to our knowledge, is the first report on BCC, SCC and VDR polymorphisms in the Spanish population.

Our data and samples were drawn from a well-established observational case-control study in which all skin cancers were histologically confirmed. The polymorphisms were analysed by one laboratory with high validity and repeatability. In fact, the total correlation was demonstrated by measuring the samples in duplicate.

## 5. Conclusions

Our results suggest that BCC and SCC are related to age and gender and that, at the same time, these variables may be influenced as well by *BsmI* and *ApaI* polymorphisms.

Further studies with a larger number of participants, namely a cohort study, and more information about possible confounding factors and vitamin D status is needed to confirm or reject our results.

## Figures and Tables

**Table 1:** Vitamin D receptor (VDR) genotype, gender, age and skin cancer risk.

Factors	Controls (%)	BCC			SCC		
		Cases (%)	OR ^1^	*p*-Value	Cases (%)	OR ^1^	*p*-Value
***BsmI***							
GG	22 (30.10)	23 (37.70)	-	0.57 ^2^	10 (50)	-	0.22 ^3^; 0.61 ^4^
AA	11 (15.10)	10 (16.40)	-		3 (15)	-	
AG	40 (54.80)	28 (45.90)	-		7 (35)	-	
***BsmI* homozygotes vs. heterozygotes**							
GG/AA	33 (45.20)	33 (54.10)	-	0.30 ^2^	13 (65)	-	0.12 ^3^; 0.39 ^4^
AG	40 (54.80)	28 (45.90)	-		7 (35)	-	
***ApaI***						-	
TT	17 (23.30)	16 (26.20)	-	0.26 ^2^	6 (30)	-	0.76 ^3^; 0.78 ^4^
GG	13 (17.80)	17 (27.90)	-		4 (20)	-	
GT	43 (58.90)	28 (45.90)	-		10 (50)	-	
***ApaI* homozygotes vs. heterozygotes**							
TT/GG	30 (41.10)	33 (54.10)	-	0.13 ^2^	10 (50)	-	0.48 ^3^; 0.75 ^4^
GT	43 (58.90)	28 (45.90)	-		10 (50)	-	
**Gender**						-	
Male	22 (30.10)	41 (67.20)	4.38 (1.93–9.90)	0.00 ^1^; 0.00 ^2^	9 (45)	-	0.21 ^3^; 0.08 ^4^
Female	51 (69.90)	20 (32.80)	-		11 (55)	-	
**Age (68.22 ± 14.53)**							
≤70 age	57 (78.10)	18 (29.50)	0.12 (0.05–0.28)	0.00 ^1^; 0.00 ^2^	4 (20)	0.07 (0.02–0.24)	0.00 ^1^; 0.00 ^3^; 0.41 ^4^
>70 age	16 (21.90)	43 (70.50)	-		16 (80)	-	

Data expressed as frequencies (percentages) and OR (IC 95%). Abbreviations: OR: Odds Ratio; BCC: Basal Cell Carcinoma; SCC: Squamous Cell Carcinoma; GG, AA, AG, TT, GT: *BsmI* and *ApaI* polymorphisms; ^1^ OR and *p*-value have been obtained through multinomial logistic regression. Only the OR, 95% CI and *p*-value data that have been significant in the model are detailed. The reference category is “Control Group”; ^2^ The *p*-value (χ^2^ test) compares the cases and the controls for BCC; ^3^ The *p*-value (χ^2^ test) compares the cases and the controls for SCC; ^4^ The *p*-value (χ^2^ test) compares the BCC cases and the SCC cases.

**Table 2:** *BsmI* and *ApaI* polymorphisms for stratum gender and skin cancer risk.

Genotype	Controls (%)	BCC			SCC		
		Cases (%)	OR ^1^	*p*-Value	Cases (%)	OR ^1^	*p*-Value
*BsmI* * Gender							
**GG**							
Male	7 (31.80)	19 (82.60)	7.01 (2.35–20.92)	0.00 ^1^	5 (50)	-	-
Female	15 (68.20)	4 (17.40)	-		5 (50)	-	
**AA**							
Male	6 (54.40)	6 (60)	-	-	3 (100)	-	-
Female	5 (45.50)	4 (40)	-		0 (0)	-	
**AG**							
Male	9 (22.50)	16 (57.10)	4.59 (1.60–13.18)	0.01 ^1^	1 (14.3)	-	-
Female	31 (77.50)	12 (42.90)	-		6 (85.7)	-	
*ApaI* * Gender							
**TT**							
Male	9 (52.90)	9 (56.30)	3.40 (1.06–10.87)	0.04 ^1^	5 (83.3)	-	-
Female	8 (47.10)	7 (43.80)	-		1 (16.7)	-	
**GG**							
Male	4 (30.80)	14 (82.40)	11.90 (3.19–44.37)	0.00 ^1^	2 (50)	-	-
Female	9 (69.20)	3 (17.60)	-		2 (50)	-	
**GT**							
Male	9 (20.90)	18 (64.30)	6.80 (2.34–19.75)	0.00 ^1^	2 (20)	-	-
Female	34 (79.10)	10 (35.70)	-		8 (80)	-	

Data expressed as frequencies (percentages) and OR (IC 95%). Abbreviations: OR: Odds Ratio; BCC: Basal Cell Carcinoma; SCC: Squamous Cell Carcinoma; GG, AA, AG, TT, GT: *BsmI* and *ApaI* polymorphisms. ^1^ OR and *p*-value have been obtained through multinomial logistic regression. Only the OR, 95% CI and *p*-value data that have been significant in the model are detailed. The reference category is “Control Group”. [*BsmI* polymorphism] * [Gender] and [*ApaI* polymorphism] * [Gender] are the factors input into the multivariate logistic regression model.

**Table 3:** Age and gender stratification and skin cancer risk.

Age	Gender	Controls (%)	BCC			SCC		
			Cases (%)	OR ^1^	*p*-Value	Cases (%)	OR ^1^	*p*-Value
≤70 age	Male	15 (26)	11 (61)	-	0.00 ^1^; 0.01 ^2^	4 (100)	-	0.00 ^3^; 0.13 ^4^
	Female	42 (73.70)	7 (38.90)	0.12 (0.04–0.37)		0 (0)	-	
>70 age	Male	7 (43.80)	30 (69.80)	-	0.07 ^2^	5 (31.30)	-	0.46 ^3^; 0.01 ^4^
	Female	9 (56.30)	13 (30.20)	-		11 (68.80)	-	

Data expressed as frequencies (percentages) and OR (IC 95%). Abbreviations: OR: Odds Ratio; BCC: Basal Cell Carcinoma; SCC: Squamous Cell Carcinoma. ^1^ OR and *p*-value have been obtained through multinomial logistic regression. Only the OR, 95% CI and *p*-value data that have been significant in the model are detailed. The reference category is “Control Group”. ^2^ The *p*-value (χ^2^ test) compares the cases and the controls for BCC. ^3^ The *p*-value (χ^2^ test) compares the cases and the controls for SCC. ^4^ The *p*-value (χ^2^ test) compares the BCC cases and the SCC cases.

**Table 4:** *BsmI* and *ApaI* polymorphisms for stratum age and skin cancer risk.

Genotype	Controls (%)	BCC			SCC		
		Cases (%)	OR ^1^	*p*-Value	Cases (%)	OR ^1^	*p*-Value
*BsmI* * Age							
**GG**							
≤70 age	17 (77.30)	7 (30.40)	0.22 (0.07–0.70)	0.01 ^1^	2 (20)	-	-
>70 age	5 (22.70)	16 (69.60)	-		8 (80)	-	
**AA**							
≤70 age	10 (90.90)	2 (20)	0.11 (0.02–0.58)	0.01 ^1^	1 (33.30)	-	-
>70 age	1 (9.10)	8 (80)	-		2 (66.70)	-	
**AG**							
≤70 age	30 (75)	9 (32.10)	0.16 (0.05–0.46)	0.00 ^1^	1 (14.30)	0.06 (0.01–0.52)	0.01 ^1^
>70 age	10 (25)	19 (67.90)	-		6 (85.70)	-	
*ApaI* * Age							
**TT**							
≤70 age	13 (76.50)	3 (18.80)	0.14 (0.03–0.60)	0.00 ^1^	3 (50)	-	-
>70 age	4 (23.50)	13 (81.30)	-		3 (50)	-	
**GG**							
≤70 age	11 (84.60)	4 (23.50)	0.21 (0.05–0.86)	0.03 ^1^	0 (0)	-	-
>70 age	2 (15.40)	13 (76.50)	-		4 (100)	-	
**GT**							
≤70 age	33 (76.70)	11 (39.30)	-	-	1 (10)	0.03 (0.00–0.30)	0.00 ^1^
>70 age	10 (23.30)	17 (60.70)	-		9 (90)	-	

Data expressed as frequencies (percentages) and OR (IC 95%). Abbreviations: OR: Odds Ratio; BCC: Basal Cell Carcinoma; SCC: Squamous Cell Carcinoma; GG, AA, AG, TT, GT: *BsmI* and *ApaI* polymorphisms. ^1^ OR and *p*-value have been obtained through multinomial logistic regression. Only the OR, 95% CI and *p*-value data that have been significant in the model are detailed. The reference category is “Control Group”. [*BsmI* polymorphism] * [Age] and [*ApaI* polymorphism] * [Age] are the factors input into the multivariate logistic regression model.

**Table 5:** The combined *BsmI*/*ApaI* genotypes in BCCs, SCCs and controls.

Genotype	Controls (%)	BCC (%)	SCC (%)	Total (%)
AAGG	1 (1.40)	0 (0)	0 (0)	1 (6)
AAGT	2 (2.70)	0 (0)	0 (0)	2 (1.30)
AATT	8 (11)	10 (16.40)	3 (15)	21 (13.60)
AGGG	1 (1.40)	0 (0)	0 (0)	1 (0.60)
AGGT	31 (42.50)	22 (36.10)	6 (30)	59 (38.30)
AGTT	8 (11)	6 (9.80)	1 (5)	15 (9.70)
GGGG	11 (15.10)	17 (27.90)	4 (20)	32 (20.80)
GGGT	10 (13.70)	6 (9.80)	4 (20)	20 (13)
GGTT	1 (1.40)	0 (0)	2 (10)	3 (1.90)

Data expressed as frequencies (percentages). Abbreviations: BCC: Basal Cell Carcinoma; SCC: Squamous Cell Carcinoma. Genotypes are expressed from their allelic combinations.

## References

[1] Giovannucci E. (2005). The epidemiology of vitamin D and cancer incidence and mortality: A review (United States). Cancer Causes Control.

[2] Holick M.F. (2004). Vitamin D: Importance in the prevention of cancers, type 1 diabetes, heart disease, and osteoporosis. Am. J. Clin. Nutr..

[3] Holick M.F. (2008). Vitamin D and sunlight: Strategies for cancer prevention and other health benefits. Clin. J. Am. Soc. Nephrol..

[4] Lomas A., Leonardi-Bee J., Bath-Hextall F. (2012). A systematic review of worldwide incidence of nonmelanoma skin cancer. Br. J. Dermatol..

[5] International Agency for Research on Cancer Working Group (2008). Vitamin D and Cancer.

[6] Reichrath J. (2006). The challenge resulting from positive and negative effects of sunlight: How much solar UV exposure is appropriate to balance between risks of vitamin D deficiency and skin cancer?. Prog. Biophys. Mol. Biol..

[7] Van der Pols J.C., Russell A., Bauer U., Neale R.E., Kimlin M.G., Green A.C. (2013). Vitamin D status and skin cancer risk independent of time outdoors: 11-year prospective study in an Australian community. J. Investig. Dermatol..

[8] Dehghan M., Pourahmad-Jaktaji R. (2016). The Effect of Some Polymorphisms in Vitamin D Receptor Gene in Menopausal Women with Osteoporosis. J. Clin. Diagn. Res..

[9] Hustmyer F.G., DeLuca H.F., Peacock M. (1993). ApaI, BsmI, EcoRV and TaqI polymorphisms at the human vitamin D receptor gene locus in Caucasians, blacks and Asians. Hum. Mol. Genet..

[10] Kostner K., Denzer N., Muller C.S., Klein R., Tilgen W., Reichrath J. (2009). The relevance of vitamin D receptor (VDR) gene polymorphisms for cancer: A review of the literature. Anticancer Res..

[11] Denzer N., Vogt T., Reichrath J. (2011). Vitamin D receptor (VDR) polymorphisms and skin cancer: A systematic review. Derm. -Endocrinol..

[12] Cohen J. (1988). Statistical Power Analysis for the Behavioural Science. Statistical Power Analysis for the Behavioural Science.

[13] von Elm E., Altman D.G., Egger M., Pocock S.J., Gotzsche P.C., Vandenbroucke J.P., Initiative S. (2007). The Strengthening the Reporting of Observational Studies in Epidemiology (STROBE) statement: Guidelines for reporting observational studies. Epidemiology.

[14] Brodland D.G., Zitelli J.A. (1992). Surgical margins for excision of primary cutaneous squamous cell carcinoma. J. Am. Acad. Dermatol..

[15] Gulleth Y., Goldberg N., Silverman R.P., Gastman B.R. (2010). What is the best surgical margin for a Basal cell carcinoma: A meta-analysis of the literature. Plast. Reconstr. Surg..

[16] Garcovich S., Colloca G., Sollena P., Andrea B., Balducci L., Cho W.C., Bernabei R., Peris K. (2017). Skin Cancer Epidemics in the Elderly as an Emerging Issue in Geriatric Oncology. Aging Dis..

[17] Joseph A.K., Mark T.L., Mueller C. (2001). The period prevalence and costs of treating nonmelanoma skin cancers in patients over 65 years of age covered by medicare. Dermatol. Surg..

[18] Pascual J.C., Belinchon I., Ramos J.M., Blanes M., Betlloch I. (2004). Skin tumors in patients aged 90 years and older. Dermatol. Surg..

[19] Oberyszyn T.M. (2008). Non-melanoma skin cancer: Importance of gender, immunosuppressive status and vitamin D. Cancer Lett..

[20] Scrivener Y., Grosshans E., Cribier B. (2002). Variations of basal cell carcinomas according to gender, age, location and histopathological subtype. Br. J. Dermatol..

[21] Cakir B.O., Adamson P., Cingi C. (2012). Epidemiology and economic burden of nonmelanoma skin cancer. Facial Plast. Surg. Clin. N. Am..

[22] Duarte A.F., Sousa-Pinto B., Freitas A., Delgado L., Costa-Pereira A., Correia O. (2018). Skin cancer healthcare impact: A nation-wide assessment of an administrative database. Cancer Epidemiol..

[23] English D.R., Armstrong B.K., Kricker A., Fleming C. (1997). Sunlight and cancer. Cancer Causes Control.

[24] Ravanat J.L., Douki T., Cadet J. (2001). Direct and indirect effects of UV radiation on DNA and its components. J. Photochem. Photobiol. B.

[25] Hutchinson P.E., Osborne J.E., Lear J.T., Smith A.G., Bowers P.W., Morris P.N., Jones P.W., York C., Strange R.C., Fryer A.A. (2000). Vitamin D receptor polymorphisms are associated with altered prognosis in patients with malignant melanoma. Clin. Cancer Res..

[26] Colston K., Colston M.J., Feldman D. (1981). 1,25-dihydroxyvitamin D3 and malignant melanoma: The presence of receptors and inhibition of cell growth in culture. Endocrinology.

[27] Tang J.Y., Xiao T.Z., Oda Y., Chang K.S., Shpall E., Wu A., So P.L., Hebert J., Bikle D., Epstein E.H. (2011). Vitamin D3 inhibits hedgehog signaling and proliferation in murine Basal cell carcinomas. Cancer Prev. Res. (Phila. PA).

[28] Raimondi S., Johansson H., Maisonneuve P., Gandini S. (2009). Review and meta-analysis on vitamin D receptor polymorphisms and cancer risk. Carcinogenesis.

[29] Gnagnarella P., Pasquali E., Serrano D., Raimondi S., Disalvatore D., Gandini S. (2014). Vitamin D receptor polymorphism FokI and cancer risk: A comprehensive meta-analysis. Carcinogenesis.

[30] Li C., Liu Z., Wang L.E., Gershenwald J.E., Lee J.E., Prieto V.G., Duvic M., Grimm E.A., Wei Q. (2008). Haplotype and genotypes of the VDR gene and cutaneous melanoma risk in non-Hispanic whites in Texas: A case-control study. Int. J. Cancer.

[31] Orlow I., Reiner A.S., Thomas N.E., Roy P., Kanetsky P.A., Luo L., Paine S., Armstrong B.K., Kricker A., Marrett L.D. (2016). Vitamin D receptor polymorphisms and survival in patients with cutaneous melanoma: A population-based study. Carcinogenesis.

[32] Santonocito C., Capizzi R., Concolino P., Lavieri M.M., Paradisi A., Gentileschi S., Torti E., Rutella S., Rocchetti S., Di Carlo A. (2007). Association between cutaneous melanoma, Breslow thickness and vitamin D receptor BsmI polymorphism. Br. J. Dermatol..

[33] Carless M.A., Kraska T., Lintell N., Neale R.E., Green A.C., Griffiths L.R. (2008). Polymorphisms of the VDR gene are associated with presence of solar keratoses on the skin. Br. J. Dermatol..

[34] Han J., Colditz G.A., Hunter D.J. (2007). Polymorphisms in the MTHFR and VDR genes and skin cancer risk. Carcinogenesis.

[35] Kostner K., Denzer N., Koreng M., Reichrath S., Graber S., Klein R., Tilgen W., Vogt T., Reichrath J. (2012). Association of genetic variants of the vitamin D receptor (VDR) with cutaneous squamous cell carcinomas (SCC) and basal cell carcinomas (BCC): A pilot study in a German population. Anticancer Res..

[36] Lesiak A., Norval M., Wodz-Naskiewicz K., Pawliczak R., Rogowski-Tylman M., Sysa-Jedrzejowska A., Sobjanek M., Wlodarkiewicz A., Narbutt J. (2011). An enhanced risk of basal cell carcinoma is associated with particular polymorphisms in the VDR and MTHFR genes. Exp. Dermatol..

[37] Von Schuckmann L., Law M.H., Montgomery G.W., Green A.C., JC V.D.P. (2016). Vitamin D Pathway Gene Polymorphisms and Keratinocyte Cancers: A Nested Case-Control Study and Meta-Analysis. Anticancer Res..

[38] Lin Y., Chahal H.S., Wu W., Cho H.G., Ransohoff K.J., Dai H., Tang J.Y., Sarin K.Y., Han J. (2017). Association between genetic variation within vitamin D receptor-DNA binding sites and risk of basal cell carcinoma. Int. J. Cancer.

[39] Carmena-Ramon R., Mateu-Puchades A., Santos-Alarcon S., Lucas-Truyols S. (2017). Actinic keratosis: New concept and therapeutic update. Aten Primaria.

[40] Burns E.M., Guroji P., Ahmad I., Nasr H.M., Wang Y., Tamimi I.A., Stiefel E., Abdelgawwad M.S., Shaheen A., Muzaffar A.F. (2017). Association of Vitamin D Receptor Polymorphisms with the Risk of Nonmelanoma Skin Cancer in Adults. JAMA Dermatol..

[41] Jorgensen T.J., Ruczinski I., Yao Shugart Y., Wheless L., Berthier Schaad Y., Kessing B., Hoffman-Bolton J., Helzlsouer K.J., Kao W.H., Francis L. (2012). A population-based study of hedgehog pathway gene variants in relation to the dual risk of basal cell carcinoma plus another cancer. Cancer Epidemiol..

